# Characteristics of Microbial Communities and Their Correlation With Environmental Substrates and Sediment Type in the Gas-Bearing Formation of Hangzhou Bay, China

**DOI:** 10.3389/fmicb.2019.02421

**Published:** 2019-10-23

**Authors:** Tao Yu, Meng Zhang, Da Kang, Shuang Zhao, Aqiang Ding, Qiujian Lin, Dongdong Xu, Yi Hong, Lizhong Wang, Ping Zheng

**Affiliations:** ^1^Department of Environmental Engineering, College of Environmental & Resource Sciences, Zhejiang University, Hangzhou, China; ^2^Advanced Environmental Biotechnology Centre, Nanyang Environment & Water Research Institute, Nanyang Technological University, Singapore, Singapore; ^3^Key Laboratory of Offshore Geotechnics and Material of Zhejiang Province, College of Civil Engineering and Architecture, Zhejiang University, Hangzhou, China; ^4^Department of Environmental Science, College of Resources and Environmental Science, Chongqing University, Chongqing, China

**Keywords:** shallow gas, gas bearing formation, microbial community, environmental substrates, sediment type

## Abstract

Shallow gas is a kind of natural gas buried in shallow strata, generally, with methane as the main component, endowing it a potential energy resource while also a potential risk to the safety of ground engineering and environment. Microbial activity is usually regarded as an important driving force to generate shallow gas via metabolizing the environmental substrates. Therefore, the research on the microbial communities will be helpful to reveal the distribution of shallow gas in the gas-bearing formation. In this study, 30 sediment samples below the seabed in Hangzhou Bay (China) from depths of 1.5 m to 55 m were collected to investigate their microbial community, environmental characteristics and sediment type (clay or sand). It turned out that the presence of shallow gas had a good correlation with the distribution of archaea rather than bacteria, with the dominant microbe of *Bathyarchaeota*, *Thaumarchaeota*, and *Euryarchaeota* in the formation. *Methanosarcinaceae* and ANME-1a with the capacity of methane metabolism occupied high proportions. The correlation analysis and redundancy analysis (RDA) suggested that ammonium was a key environmental substrate to indicate the microbial community in the formation. The sediment type was proposed to shape environmental substrates in the formation, thus further affecting the microbial communities. The clay strata were demonstrated to have an important role in the generation and distribution of shallow gas, and more attention should be paid in terms of its resource discovery and engineering safety assessment.

## Introduction

Shallow gas, with methane as the main component, is a kind of natural gas buried in shallow formation (within the topmost 1000 m of sediment) (Davis, 1992; Roy et al., 2019). Its reserves are plentiful globally. There are many gas reservoirs with exploitation value in the Black Sea (Xing and Spiess, 2015) and Arctic Ocean (Rajan et al., 2013). However, due to the shallow distribution in the formation, shallow gas is easy to be affected by the disturbance of environment and thus release from the sediment, resulting in the risks of eruption and leakage (Gentz et al., 2014; Vielstädte et al., 2015), and engineering safety (Xu et al., 2017). On the other hand, methane is reported as a potent greenhouse gas with the warming potential of 20–40 times that of carbon dioxide (Shindell et al., 2009; Zhang et al., 2019). Therefore, the released shallow gas with high content of methane will potentially intensify the greenhouse effect.

Microorganisms are regarded as an important driving force to form shallow gas, especially in the marginal seas, e.g., estuary and coastal zone, which are characterized by high nutrient inputs from the land (Liu et al., 2015). Nitrogen- and sulfur-bearing organic matter (OM) is typically abundant in estuarine sediments (Wakeham et al., 1997; Burdige, 2005), and could be further converted to shallow gas by microorganisms in the strata. According to previous research, archaea are believed to play an important role in the substrate conversion in the marine sediment. For example, *Bathyarchaeota* are widespread in marine sediments (Meng et al., 2014), and can metabolize complex OM (Lloyd et al., 2013; Yu et al., 2018). *Thaumarchaeota* are closely related to nitrogen metabolism, and are more abundant in the marine sediment. Note that, most of the newly reported ammonia oxidizing archaea (AOA) belong to this phylum (Santoro et al., 2015). *Euryarchaeota* contain the largest group of methanogens, and are capable of converting OM to methane gas (Lang et al., 2015). While the anaerobic methanotrophic (ANME) were reported to couple with sulfate reducing bacteria (SRB), and could anaerobically oxidize methane to low molecular weight OMin marine sediments (Hinrichs et al., 1999; Holler et al., 2011; Wang et al., 2014; Wegener et al., 2015). Additionally, bacteria, such as *Proteobacteria*, *Firmicutes*, *Actinobacteria*, *Marinobacter*, and *Alcanivorax*, are also reported to be involved in the transformation of substrates in the marine sediment (Kimes et al., 2014; Kleindienst et al., 2015; Zhang et al., 2015).

Previous studies on microbial communities in sediments usually focused on the shallow surface within a single sediment type (Hu et al., 2014; Yu et al., 2018). However, the deep sediments had been revealed with several different types of sediment layers, where the microbial communities were quietly diverse (Inagaki et al., 2003; Nunoura et al., 2016). The sediment type in the vertical dimension is also an important factor that can affect the distribution of both substrate and shallow gas in the formation, leading to a different microbial structure.

The Hangzhou Bay is the largest estuary in China, and receives a lot of terrestrial substrates from the Yangtze River and the Qiantang River (QR) every year (Xu, 2016). Since the last glaciation, with the rise and fall of sea level, the QR incised valley underwent three stages: (1) deep-cutting stage; (2) rapid-filling stage; and (3) burial stage. Due to the variation of sedimentary environment, quite different rock strata such as sand and clay deposited vertically in the QR incised valley (Lin et al., 2005; Zhang et al., 2013). Because of the rapid deposition rate (2–3 cm/a) (Lin et al., 2005) and rich substrate in the sediment, the gas reservoirs are shallow and widespread, and thus provide a typical case to investigate the relationship among the microbial community, environmental substrates and sediment type.

In the present work, the sediment samples below seafloor in a range of 0–35 m were collected near Yushan Island, Hangzhou Bay, China. Based on the methane content *in situ* (main gas component with a proportion over 95%), the strata were divided into gas-bearing layer and gas-free layer. The microbial community, environmental substrates and sediment type in both layers were clarified. A comparison analysis among the microbial community, environmental substrate and sediment type in the gas-bearing layer was carried out. The information gathered in the present work demonstrated the core role of microbe in the formation of shallow gas and revealed the unique microbial structure in the gas-bearing layer.

## Materials and Methods

### Collection of Samples

**FIGURE 1:**
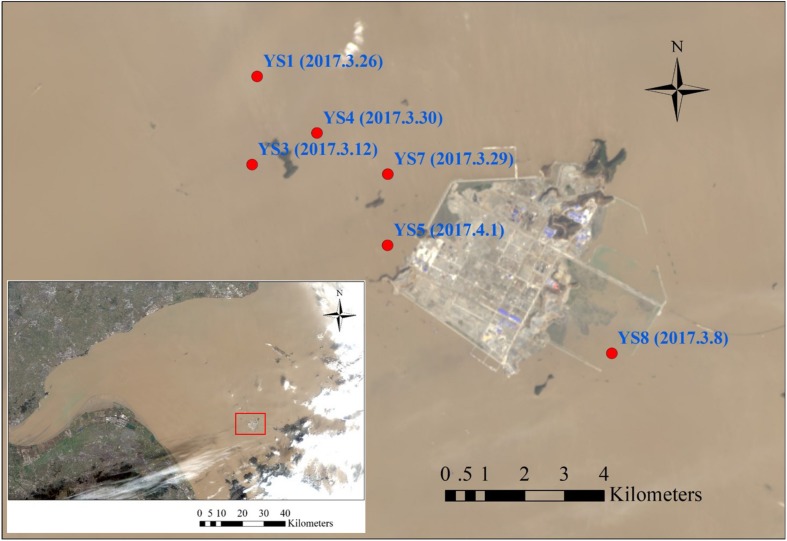
The location of sampling sites.

A total of 30 samples were collected from six sites around the Yushan Island (Hangzhou Bay) of Zhejiang Province, China, in spring 2017. The detail information of sampling sites was shown in Figure 1 and Supplementary Table S1. The samples were collected vertically through drilling, and the drill core at each site was collected. Each sample was divided into two parts, one was stored at 4°C for microbial community analysis and inorganic nitrogen measurement, the other was air-dried for the physicochemical analysis.

### Analysis of Environmental Substrates

Ammonium, nitrite, and nitrate were extracted from wet sediments using 2 M KCl (Shen et al., 2013; Hu et al., 2014), and determined according to the standard methods (APHA, 2017). Air-dried sediment samples were sifted for the analysis of other physical and chemical properties (passed through an 18-mesh sieve for the determination of moisture and sulfate, passed through a 100-mesh sieve for the determination of OM and nutrient elements). The moisture of air-dried sediment was determined by the stoving method. The content of soil OM was determined by the K_2_Cr_2_O_7_ oxidation method. Sulfate was extracted by water (the water-soil ratio was 5:1) and was determined by ion chromatography (DIONEX ICS-1000, America). The content of total carbon (TC), nitrogen (TN), and sulfur (TS) was determined using the elemental analyzer (elementar vairo EL CUBE, Germany).

### Measurement of Methane Content

The methane content of sediment samples was determined using Static Head Space Method. Intact core was collected immediately using ring-knife when the sediment column was taken out. 3.0 mL sediment samples were put into serum bottles with 20 mL headspace. 6 mL NaOH (1 M) were added into the serum bottle to inhibit methanogenesis. The serum bottles were sealed with butyl rubber stoppers. They were oscillated at 25°C until equilibrium. The methane content in the headspace was determined with Trace GC Ultra gas chromatograph (Thermo Fisher Scientific, United States). The methane content of sediments was calculated using the following equation:

$$\left[{\text{CH}}_{4}\right]=\frac{c\,\left({\text{CH}}_{4}\right)\,V_{\text{headspace}}}{V_{\text{sediment}}}$$

where c(CH_4_) is the methane concentration in the headspace (in μg/mL), V_headspace_ is the volume of the serum bottle headspace (mL) after the adding of sediment and NaOH, and V_sediment_ is the volume (mL) of sediment added to the serum bottle. The temperature was 25°C, and the pressure in the serum bottle was assume as one bar (Johnson et al., 1990; Lloyd et al., 2011; Li et al., 2012).

### DNA Extraction and Sequencing

Total DNA was extracted using a FastDNA SPIN kit for soil (MP biochemicals, United States) according to the manufacturer’s protocol from 0.25 g sediments. Then, 16S rRNA genes were amplified using the bacterial and archaeal primer sets 338F (5′-ACTCCTACGGGAGGCAGCAG-3′)/806R (5′-GGACTACHVGGGTWTCTA AT-3′) and Arch344F (5′-ACGGGGYGCAGCAGGCGCGA-3′)/Arch915R (5′-GTGCTCCCCCGC CAATTCCT-3′). The product of PCR was examined using 2.0% agarose gel electrophoresis, and the purification recovery was conducted on the targeted strips which appeared in the agarose gel using SanPrep Column PCR Product Purification Kit (Sangon Biotech., Shanghai, China). The purified 16S rRNA gene amplicons were sequenced on the Illumina MiSeq platform by MajorBio, Shanghai. Quality control, sequences clustering and OTU taxonomic annotation (Silva database) were completed on I-Sanger Bioinformatics cloud platform^1^. In addition, α-diversity indexes including Sobs (number of OTU observed), Ace, Chao1; Shannoneven, Simpsoneven (a Shannon/Simpson index-based measure of evenness); Shannon and Simpson were calculated on that platform. The gene sequences obtained in this study are available in NCBI: PRJNA541126 and PRJNA542038.

### Statistical Analyses

**FIGURE 2:**
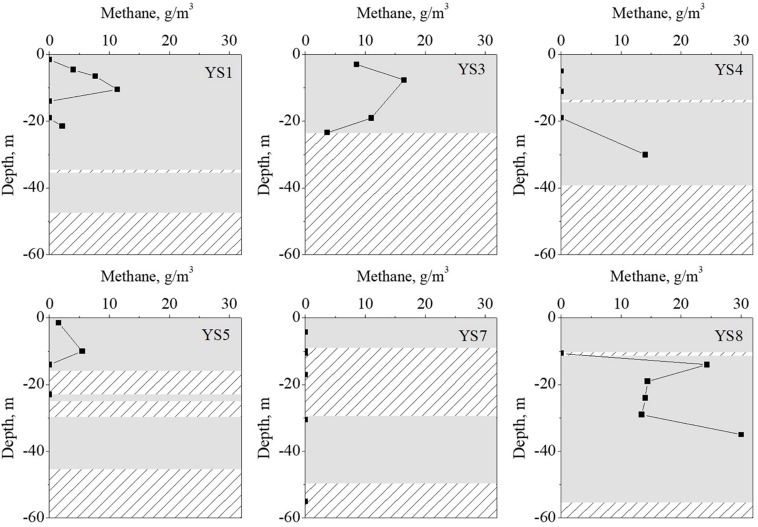
The methane content and sediment type of strata with difference depth. The gray rectangles represent the clay strata, rectangles filled with lines represent the sand strata.

Strata were divided into gas-bearing layer and gas-free layer based on the methane content. Meanwhile, they were divided into clay stratum and sand stratum according to their texture (Figure 2). *T*-test (SPSS, 22.0) was used to compare the differences of the microbial community (α-diversity indexes and relative abundance of dominant groups) and environmental factors in different strata. The correlation analysis was completed with the *corrplot* R package. Redundancy analysis (RDA) was used to explore the relationship between response variables (i.e., relative abundance of archaea at family level or bacteria at genus level) and explanatory variables (methane and environmental substrates such as OM, Ammonia-N). RDA was calculated with the *vegan* package and plotted using the *ggplot2* package. Chi-square test of shallow gas and sediment type was completed with function Mosaic in the *vcd* package and showed as Mosaic plot. Other plots were completed using software Origin (V 8.0).

## Results

### Methane and Environmental Substrates in the Formation

**FIGURE 3:**
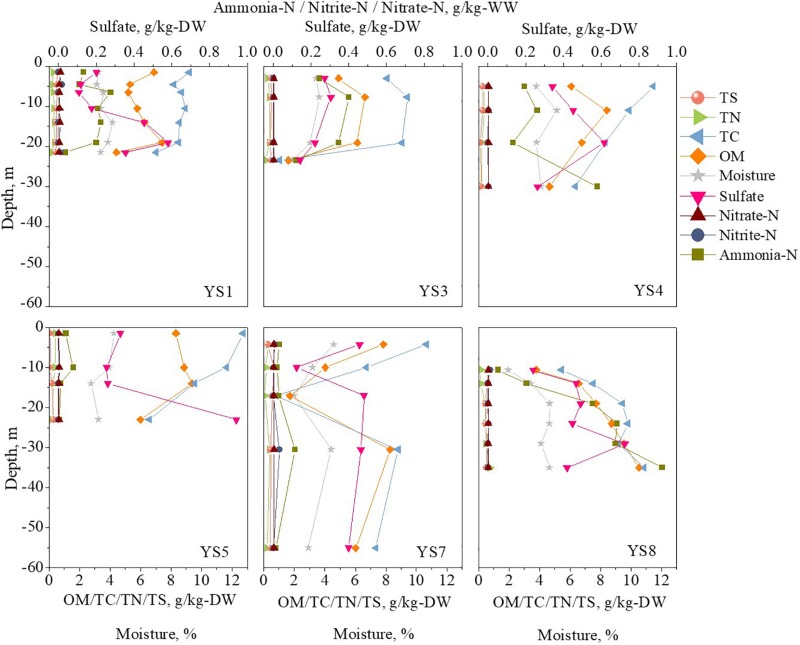
The vertical profiles of environmental factors (Data were shown in Supplementary Table S2).

The vertical distribution of the methane in the sediment cores are shown in Figure 2. The methane concentrations were in the range of 0–30.1 g/m^3^, which were similar to the values reported in previous literature (Lloyd et al., 2011; Li et al., 2012; Hu et al., 2014; Tong et al., 2017). The environmental substrates of the sediment samples were determined, and the results are shown in Figure 3 (detail information can be found in Supplementary Table S2). The OM was the dominant substrate in the sediments, and was in a range of 1.61–10.52 g/kg-DW (dry weight) with 6.45 g/kg-DW on average. Ammonia-N was the main form of inorganic nitrogen with contents of 0.004–0.92 g/kg-WW (wet weight) and 0.23 g/kg-WW on average. The contents of nitrite-N and nitrate-N were much lower than that of ammonia-N, and even below the detect limitation. Hygroscopic water content (moisture) was in a range of 1.85–5.13% with 3.60% on average. Further analysis was carried out to reveal the correlation among the environmental substrates and the results (shown in Supplementary Figure S1) demonstrated a significantly positive correlation between ammonia-N, TC, TN, moisture and OM. It is worth noting that the correlation between TN and OM (*r* = 0.81) was stronger than that between ammonia-N and OM (*r* = 0.42). It should be noted that methane was positively correlated with ammonia-N (*r* = 0.8), but not with OM (*r* = 0.3).

### Microbial Communities in the Formation

**FIGURE 4:**
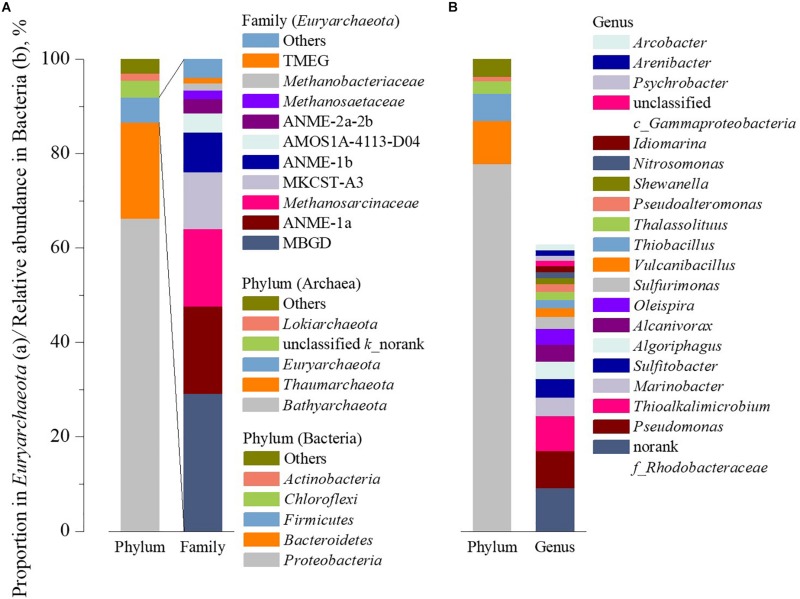
Community composition of archaea **(A)** and bacteria **(B)** in the sediments.

The microbial communities of the sediment samples were analyzed using 16S rRNA gene high-throughput sequencing, and the results are showed in Figure 4. In the archaeal domain, 17 phyla were detected, *Bathyarchaeota*, *Thaumarchaeota*, and *Euryarchaeota* were the dominant phyla with average relative abundance of 66.34, 20.37, and 5.29%, respectively (Figure 4A). *Bathyarchaeota* were the most abundant in 26 samples, while *Thaumarchaeota* were detected as the dominant microbe in the samples from deep of site YS8 (Supplementary Figure S2a). At the class level, *Bathyarchaeota*, a novel discovered branch in the archaeal domain, cannot be further identified due to lack of reference. Group C3 were the dominant class in *Thaumarchaeota* (Supplementary Figure S2a). The distinguishability of *Euryarchaeota* was better than that of the first two phyla. At the class level, *Methanomicrobia* or *Thermoplasmata* were the dominant class, and could be further identified as Marine Benthic Group D (MBGD), ANME-1a, *Methanosarcinaceae*, MKCST-A3 and ANME-1b at the family level with relative abundances of 29.18, 18.49, 16.43, 11.95, and 8.51%, respectively.

In the bacterial domain, 128 genera affiliated into 44 phyla were detected. *Proteobacteria*, *Bacteroidetes*, *Firmicutes*, *Chloroflexi*, and *Actinobacteria* were dominant phyla with average relative abundances of 77.88, 9.13, 5.74, 2.65, and 0.95%, respectively (Figure 4B). At genus level, *Pseudomonas*, *Thioalkalimicrobium*, *Marinobacter*, and *Sulfitobacter* were the top 4 genera, with average relative abundances of was 7.96, 7.31, 3.96, and 3.94%, respectively. It is worth noting that 16 out of the 20 most abundant genera belong to *Proteobacteria*.

### Relationship Between Microbial Community and Shallow Gas

**FIGURE 5:**
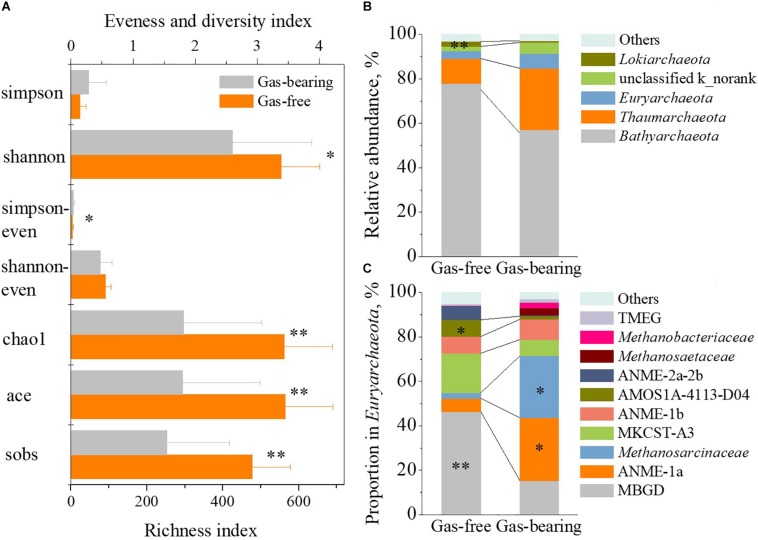
The difference of archaeal community between strata with shallow gas or not. The difference of α-diversity indexes **(A)**, relative abundance of archaeal phylum **(B)** and proportion in *Euryarchaeota* at family level **(C)** between gas-bearing layers and gas-free layers (asterisk: the level of significance. “^∗^” represents *p* < 0.05, “^∗∗^” represents *p* < 0.01).

Microorganisms are regarded as an important driving force to form shallow gas. The characteristics of the microbial community in the gas-bearing and gas-free layers was further analyzed, and the results are showed in Figure 5.

In archaeal domain, the results of α-diversity analysis (Figure 5A) showed significant lower species richness (*p* < 0.01), evenness (*p* < 0.05) and diversity (*p* < 0.05) of the archaeal community in the gas-bearing layers than those in the gas-free layers. In detail, no statistically significant differences were observed for *Bathyarchaeota*, *Thaumarchaeota*, and *Euryarchaeota* at the phylum level (Figure 5B). Note that, among the members of *Euryarchaeota*, the proportion of MBGD in gas-bearing layers was 15.19% on average, which was significantly lower than that in gas-free layers (46.41%, *p* < 0.01). The proportion of ANME-1a was 28.54% in gas-bearing layers, which was 3.7 times higher than that in gas-free layers (6.12%, *p* < 0.05). The proportion of *Methanosarcinaceae* was 27.79% in gas-bearing layers, which was 10.3 times higher than that in gas-free layers (2.46%, *p* < 0.05) (Figure 5C). Consequently, the statistical analysis results indicated a good correlation between archaeal community and shallow gas.

In the bacterial domain, the results of α-diversity analysis (Supplementary Figure S3a) showed a lower species richness (*p* < 0.05) of bacterial community in the gas-bearing layers than that in the gas-free layers. In detail, only the relative abundance of *Shewanella* in the gas-bearing layers (2.30%) at the genus level was significantly different (*p* < 0.05) from that in the gas-free layers (0.25%) (Supplementary Figure S3b). That is to say, the correlation between shallow gas and bacterial community was poor.

### Relationship Between Microbial Community, Methane and Environmental Substrates

#### Relationship Between Archaea, Methane and Environmental Substrates

**FIGURE 6:**
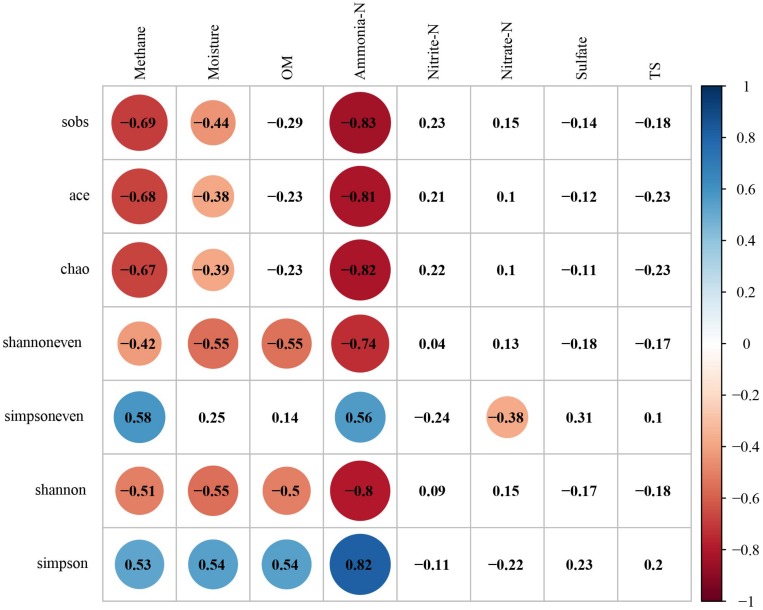
The correlation analysis between the species diversity of archaeal community, methane and environmental factors (Digits represents Pearson correlation coefficients; filled color indicates significant correlation, blue represents positive correlation, red represents negative correlation, shade of color represents strength of correlation).

It is well-known that environmental substrates have a great influence on the metabolism of microbe, and thus affect the characteristics of microbial communities in the formation. The results showed that the α-diversity of archaea was negatively correlated with multiple environmental substrates (Figure 6). The correlation between α-diversity and ammonia-N was the strongest., whereas the species richness (Sobs, *r* = −0.83, *p* < 0.01; Ace, *r* = −0.81, *p* < 0.01; Chao1, *r* = −0.82; *p* < 0.01), evenness (Shannoneven, *r* = −0.74, *p* < 0.01; Simpsoneven, *r* = 0.56, *p* < 0.01) and diversity (Shannon, *r* = −0.80, *p* < 0.01; Simpson, *r* = 0.82, *p* < 0.01) were negatively correlated with ammonia-N. α-diversity and methane had a similar but weaker correlation with α-diversity and ammonia-N. It should be noted that the species richness (Sobs, *r* = −0.69, *p* < 0.01; Ace, *r* = −0.68, *p* < 0.01; Chao1, *r* = −0.67; *p* < 0.01), evenness (Shannoneven, *r* = −0.42, *p* < 0.05; Simpsoneven, *r* = 0.58, *p* < 0.01) and diversity (Shannon, *r* = −0.51, *p* < 0.01; Simpson, *r* = 0.53, *p* < 0.01) were negatively correlated with methane.

**FIGURE 7:**
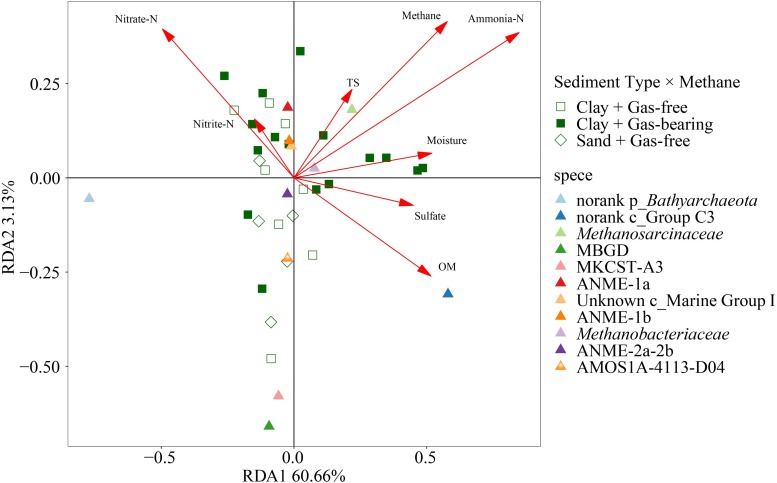
Ordination plot of redundancy analysis (RDA) for the dominant archaeal families with methane and environmental substrates as constraining variables.

The top 10 families affiliated to *Bathyarchaeota*, *Thaumarchaeota* or *Euryarchaeota* were used to analyze the variation of relative abundance of archaea in response to methane and environmental substrates by RDA (Figure 7). The Monte Carlo permutation test showed that up to 67.43% of the variance in the relative abundance of dominant archaeal families could be explained by methane and environmental substrates (with 999 permutations, *p* = 0.002). Among all the explanatory variables, ammonia-N, methane, OM, moisture and nitrate had a significant effect on the species dominance of archaeal community (Supplementary Table S3). The relative abundance of dominant groups had the strongest correlation with ammonia-N, which was consistent with the correlation analysis of α-diversity and environmental factors. As the main methanogens in the formation, *Methanosarcinaceae* was positively correlated with ammonia-N and methane.

#### Relationship Between Bacteria, Methane and Environmental Substrates

It was found that the environmental substrates had a weak influence on the α-diversity of bacterial community (Supplementary Figure S4). Among all the environmental substrates, the correlation between species diversity and ammonia-N was the strongest. The species richness (sobs, *r* = −0.57, *p* < 0.01; ace, *r* = −0.59, *p* < 0.01; chao1, *r* = −0.58; *p* < 0.01) and diversity (Shannon, *r* = −0.46, *p* < 0.05) were negatively correlated with ammonia-N significantly.

The top 20 genera were used to analyze the variation of relative abundance of bacteria in response to methane content and environmental substrates by RDA (Supplementary Figure S5). The Monte Carlo permutation test showed that up to 43.30% of the variance in the relative abundance of dominant bacterial genera could be explained by environmental factors (with 999 permutations, *p* = 0.007). Among all the explanatory variables, ammonia-N, methane and nitrate had a significant effect on the species dominance of bacterial community (Supplementary Table S4). The relative abundance of dominant groups had a strongest correlation with ammonia-N, which was consistent with the correlation analysis of α-diversity and environmental factors. It is worth noting that the sand samples were clustered into an independent group in the RDA plot.

### Relationship Between Microbial Community and Sediment Type

The type of sediment characterizes the physicochemical properties of strata and could affects the distribution of substrates and thus affects the microbial community. Based on soil particle gradation, the strata can be divided into the clay and sand strata. Their α-diversity and dominant species of the microbial community were further analyzed to reveal the influence of texture on microbial community.

**FIGURE 8:**
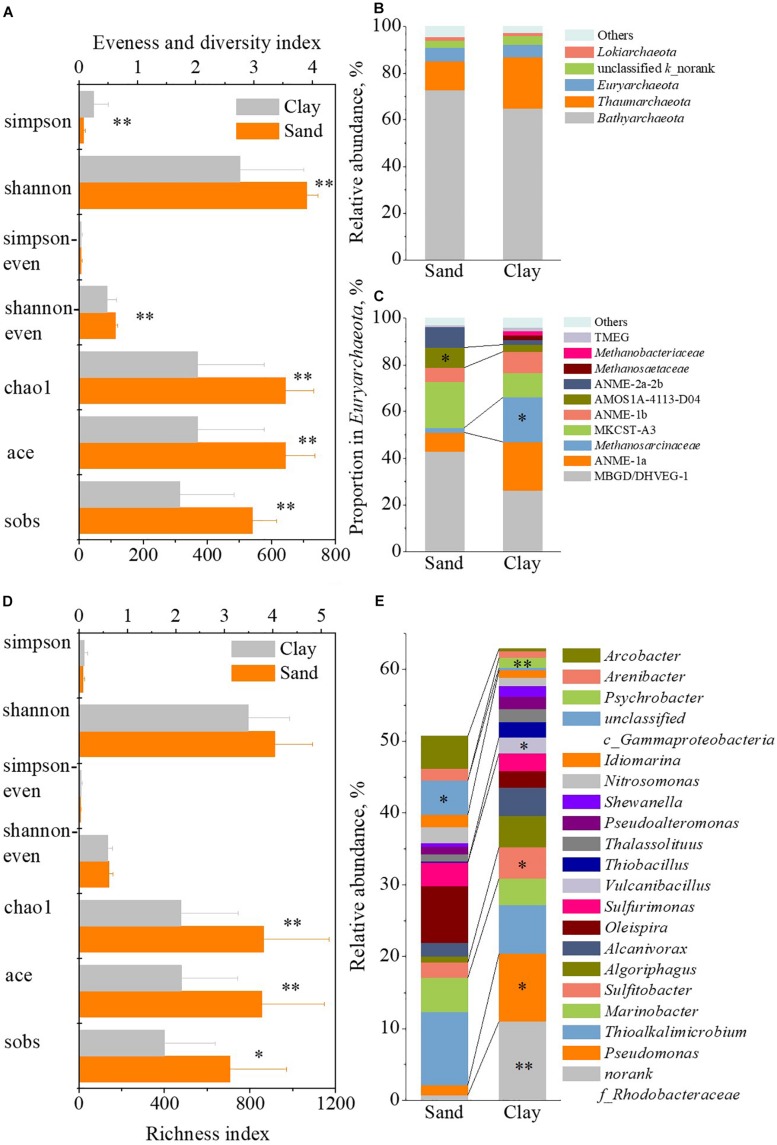
The difference of microbial community between clay strata and sand strata. The difference of α-diversity indexes of archaeal community **(A)**, relative abundance of archaeal phylum **(B)**, proportion in *Euryarchaeota* at family level **(C)**, α-diversity indexes of bacterial community **(D)**, and relative abundance of bacterial genus **(E)** between sand strata and clay strata.

As to the archaea, the analysis of α-diversity (Figure 8A) showed that the Sobs, Ace and Chao1 indexes were significantly lower (*p* < 0.01) in clay strata than those in sand strata, which indicated a lower species richness of archaea in clay strata. A significantly lower Shannoneven index (*p* < 0.01) suggested a lower species evenness of archaea in the clay strata. Meanwhile, the Shannon index was lower (*p* < 0.01), while the Simpson index was higher (*p* < 0.01) in clay strata, implied a lower species diversity of archaea. The analysis of species dominance (Figures 8B,C) showed that, among the members of *Euryarchaeota*, the proportion of *Methanosarcinaceae* in clay strata was 19.43% on average, which was 8.57 times higher than that in sand strata (2.03%, *p* < 0.05).

As to the bacteria, the analysis of α-diversity (Figure 8D) showed that the Sobs, Ace and Chao1 indexes were significantly lower (*p* < 0.05 for Sobs index; 0.01 for Ace and Chao1 index) in clay strata than those in sand strata, which suggested the lower species richness of bacteria in clay strata. The analysis of species dominance (Figure 8E) showed that the relative abundance of *Pseudomonas* (9.47% in average, *p* < 0.05), *Sulfitobacter* (4.35% in average, *p* < 0.05), *Vulcanibacillus* (2.19% in average, *p* < 0.05) and *Psychrobacter* (1.35% in average, *p* < 0.01) were significantly higher than those in sand strata (0.77, 1.34, 2.14, 0.01, and 0.04%, respectively).

## Discussion

### Characteristics of Methane and Environmental Substrates Distribution in Hangzhou Bay

The Hangzhou Bay is the largest estuary in China, and receives masses of terrestrial substrates from the Yangtze River and the QR every year (Xu, 2016). Hindered by Zhoushan Islands, the substrates are easily deposited in the bay. In this study, most of the collected sediment samples contained higher OM than the lower limit for potential terrestrial gas sources (3.1 g/kg) (Zhou et al., 1994), and only a few samples contained higher OM than the lower limit for potential marine gas sources (8.6 g/kg) (Dudley and George, 1981) (Supplementary Figure S6). Yushan Island is located at the edge of the QR incised valley, and the formation is mainly consisted of shallow marine sediments. As such, it is difficult to form large-scale shallow gas reservoirs compared with other formation dominated by floodplain sediments (Zhang et al., 2013). The accumulation of ammonium in strata indicated a weak nitrification. TN showed a close relationship with OM in the formation, which was accordant with the fact that organic nitrogen is the main form of soil nitrogen. However, the correlation between ammonia-N and OM was weak (*r* = 0.42), which might be ascribe to the hypothesis of that ammonium originated from the OMs consumed in geological period. Similarly, methane and ammonia-N (*r* = 0.8) in the formation have a stronger correlation than that of methane and OM (*r* = 0.3), which can also contribute to the hypothesis mentioned above.

As shown in Supplementary Figure S7, ammonia-N in gas-bearing layers was 0.34 g/kg-WW on average, which was significantly higher than that in gas-free layers (0.10 g/kg-WW, *p* < 0.01). Moisture, OM, TC, TN and ammonia-N in clay strata were significantly higher than those in sand strata (*p* < 0.05 for OM and *p* < 0.01 for others). These results demonstrated that sediment type had a strong influence on the environmental substrates distribution, which might be overlooked.

### Characteristics of Microbial Community in Hangzhou Bay

*Bathyarchaeota* were the dominant archaea in most of sediment samples in Hangzhou Bay, which was similar with the microbial community in other researches of marine sediments (Sorensen and Teske, 2006; Webster et al., 2014; Meador et al., 2015). *Bathyarchaeota* were widespread in natural habitats (Meador et al., 2015), especially in marine sediments (Meng et al., 2014). *Bathyarchaeota* were capable of heterotrophic metabolism (Biddle et al., 2006; Evans et al., 2015), and had been suggested to play an important role in the breakdown of complex OMs such as detrital proteins (Lloyd et al., 2013) and lignin (Yu et al., 2018).

The AOA belonging to *Thaumarchaeota* are the most well-studied archaeal group (Santoro et al., 2015), which are the main ammonia oxidizers in the marine environment (Zheng et al., 2013). However, AOA were lacking in sampling sites, which explained the accumulation of ammonium. *Euryarchaeota* contain the largest variety of methane related archaea. As a subgroup of *Euryarchaeota*, *Methanosacinaceae* were the most abundant in the formation (Supplementary Figure S2a), which were believed to act methanogenesis from methyl compounds such as methylamines and methyl sulfides. Additionally, it was found that methylotrophic methanogens incorporate the majority of dissolved inorganic carbon into lipids in marine sediment (Yin et al., 2019). By contrast, the relative abundance of hydrogenotrophic methanogens, e.g., *Methanobacteriaceae*, was negligible in the formation, which is possibly due to the low hydrogen concentration in anaerobic sediment (Kessler et al., 2019) and strong competition of SRB over hydrogen. A lot of AOM were found in the formation, and ANME-1a was the most abundant group. ANME could oxidize methane in anaerobic environment (Wang et al., 2014), which was thought to act sulfate-coupled AOM cooperating with SRB (Wegener et al., 2015).

*Proteobacteria* was the dominant bacteria in the formation, which has been confirmed in many previous studies (Inagaki et al., 2003). Followed by *Bacteroidetes* and *Firmicutes*, which were thought to be the key bacterial communities in the acidogenic process of OM (Li et al., 2019). In this study, the genera with high relative abundance such as *Pseudomonas* and *Algoriphagus* were heterotrophic. In addition, many genera, e.g., *Algoriphagus* (agar) and *Arenibacter* (alginic acids) are capable of degrading complex OMs to simple compounds. This in turn indicated that the hydrolysis of macromolecular OM might be one of the key steps for the overall carbon mineralization process in the sediment (Flury et al., 2016). SRB, e.g., *Desulfuromonas* and *Desulfuromonas*, which could utilize methanogenic substrates such as hydrogen and acetate, were also abundant in the sediment. However, the common syntrophic and fatty acid oxidizing bacteria, e.g., *Syntrophobacter* and *Desulfovibrio*, were deficient. More efforts should be devoted on this aspect in future work. Meanwhile, the relative abundance of ammonia oxidizing bacteria (AOB) was low in the formation. Only *Nitrosomonas* were abundant in several sample, e.g., 7–26 (16.11%), 7–8 (8.21%), and 5–8 (6.82%) (Supplementary Figure S2b). This result confirmed the fact that nitrification is weak in the studied strata.

Generally, the abundance of microbes with methanotrophic function, e.g., *Methanosacinaceae*, ANME-1a in *Euryarchaeota* were accordant to the distribution of shallow gas. Although the proportion of methanogen in the *Euryarchaeota* was significantly positively correlated with methane (*Methanosacinaceae*, *r* = 0.42; *Methanosaetaceae*, *r* = 0.41; *Methanobacteriaceae*, *r* = 0.44), but all were weaker than their respective correlation with ammonia-N (*Methanosacinaceae*, *r* = 0.6; *Methanosaetaceae*, *r* = 0.46; *Methanobacteriaceae*, *r* = 0.64) (Supplementary Figure S8), Meanwhile, weaker than the correlation between ammonia-N and methane (Supplementary Figure S1).

### Ammonium-a Key Environmental Substrates to Indicate the Microbial Community in Hangzhou Bay

In this work, ammonium was the most important substrate associated with microbial community in the gas-bearing formation in Hangzhou Bay. In theory, ammonium does not support microbial community directly besides being used as nitrogen source and energy source, but it can indicate the decomposition of nitrogenous OMs. In the formation, nitrogenous OMs are converted into methane, carbon dioxide (biogas) and ammonium by heterotrophic microorganisms. Due to the absence of ammonia oxidizers (see section “Characteristics of Microbial Community in Hangzhou Bay”) and the adsorption of ammonium by clay minerals, the ammonium would accumulate. In this regard, the ammonium concentration *in situ* could present the degree of heterotrophic degradation of OMs.

### Effect of Sediment Type on the Microbial Community

The sediment type may affect the distribution of shallow gas and substrates in the formation. The Chi-square test (Supplementary Figure S9) showed a close relationship between shallow gas and sediment type (*p* < 0.05), and the shallow gas was revealed to be favorable to present in the clay strata in Hangzhou Bay.

Moreover, the characteristics of sediment would have similar effect on the distribution of environmental substrates. In this regard, the sediment type could greatly affect the microbial communities. Clay strata contained more OMs and other substrates than sand strata, triggering the enrichment of *Methanosacinaceae*, resulting in a greater methanogenic potential. Additionally, heterotrophic bacteria also occupied high relative abundance in clay strata, e.g., *Pseudomonas*, *Sulfitobacter*, *Vulcanibacillus*, and *Psychrobacter*. These results illustrated that the clay strata had a great metabolic potential of OMs, and was the main gas producing layer. Therefore, the gas potential in these strata deserved more attention and test for resource discovery and engineering risk assessment.

### The Remnants of Geological Microbial Activity in the Gas-Bearing Layers

**FIGURE 9:**
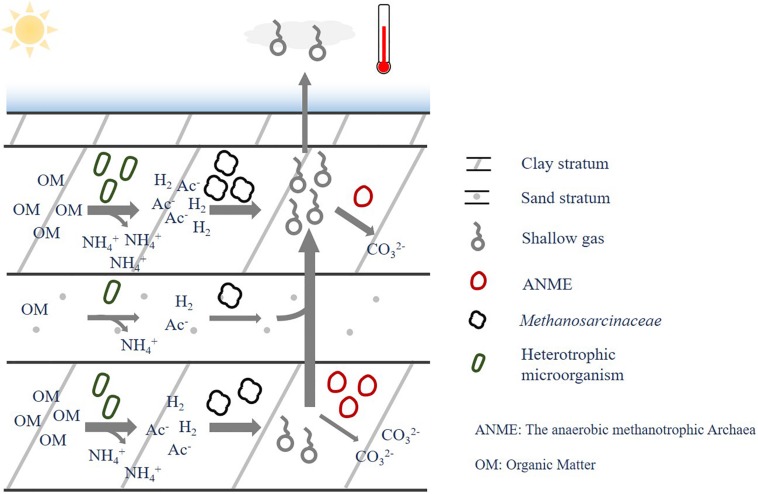
Relationship between sediment type, environmental factors, microbial communities and biogas.

To sum up, the presence of shallow gas in the gas-bearing layers is ascribed to several factors, i.e., sediment type, environmental substrates and associated microbial organisms. The microbial organism played a key role to convert the environmental substrates to shallow gas via microbial metabolism, while the sediment characteristics could shape the environmental factors in the formation, thus affecting microbial communities and shallow gas production. Methane and ammonium could be produced during the microbial degradation of OMs, where methane could be further consumed or transferred to other strata; while the ammonium was more likely to be stable and stored *in situ* in this environment. It should be noted that the iron- or manganese-dependent AOM were reported to widely distribute in the sediment (Beal et al., 2009; Sivan et al., 2011; Bar-Or et al., 2017), which could be a potential methane sink to elevate the methane consumption rate in the *in situ* strata of methane production. More research is proposed to carry out to verify their effects on the correlations of methane with other factors. Consequently, the correlation of methanogen, methane with ammonium were stronger than that between methanogen and methane, and no close relationship was found between microbial community, methane and existing OMs (Figure 9).

## Conclusion

In Hangzhou Bay, it was found that the presence of shallow gas had a good correlation with the distribution of archaea while a weak correlation with bacteria. The dominant archaeal microorganisms were *Bathyarchaeota*, *Thaumarchaeota*, and *Euryarchaeota*. The dominant bacteria were *Proteobacteria*, *Bacteroidetes*, *Firmicutes*, *Chloroflexi*, and *Actinobacteria*, and the dominant genera, such as *Pseudomonas*, were mostly heterotrophic. The gas-bearing layers were revealed with lower species richness, evenness and diversity of archaeal community than those in gas-free layers, while the proportion of *Methanosacinaceae* and ANME-1a in *Euryarchaeota* in gas-bearing layers was higher than that in gas-free layers. Ammonium was supposed to be a vital environmental substrate to indicate the characteristics of the microbial community in the formation, and a higher content of ammonium suggested a lower α-diversity of the microbial community and a higher proportion of methanogenic microorganisms. The sediment type could affect the distribution of shallow gas through shaping environmental substrates and the microbial communities. The clay strata were illustrated to have a higher abundance of archaea and be easier for accumulation of shallow gas than sand strata.

## Data Availability Statement

The datasets generated for this study can be found in the NCBI, PRJNA541126, PRJNA542038.

## Author Contributions

TY and MZ: drafting the work. DK and SZ: acquisition and analysis of data for the work. AD, QL, and DX: revising the manuscript. YH and LW: interpretation of data for the work. PZ: final approval of the version.

## Conflict of Interest

The authors declare that the research was conducted in the absence of any commercial or financial relationships that could be construed as a potential conflict of interest.

## Footnotes

1 https://www.i-sanger.com
