# Supplementary material for: Characteristics of Microbial Communities and Their Correlation With Environmental Substrates and Sediment Type in the Gas-Bearing Formation of Hangzhou Bay, China
Source: Front Microbiol. 2019 Oct 23;10:2421. doi: 10.3389/fmicb.2019.02421 (PMC6819322; doi:10.3389/fmicb.2019.02421)
Supplement: Supplementary file 1 [file Table_1.DOCX]

Supplementary Material

# Supplementary Figures and Tables

## Supplementary Figures


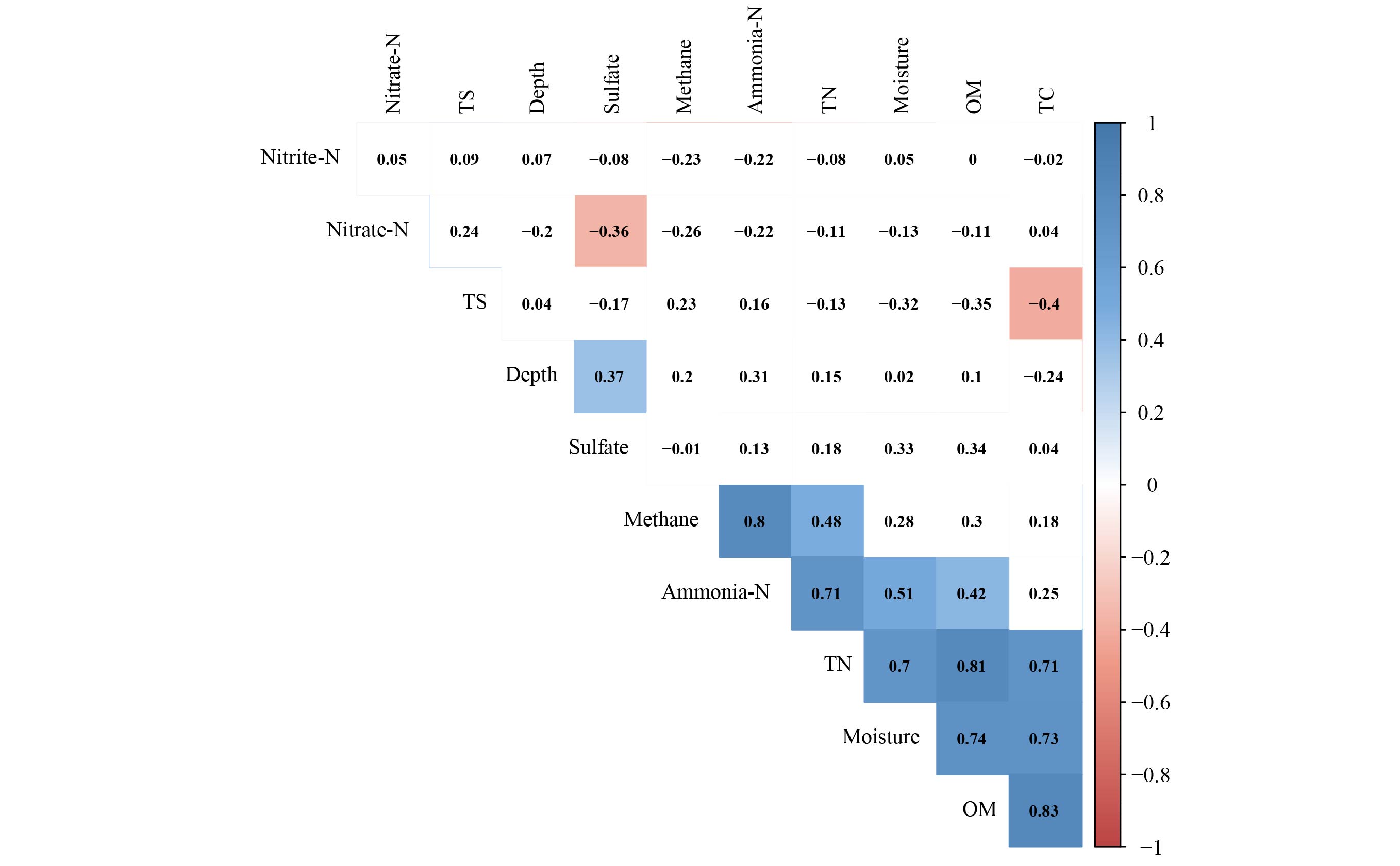


**Supplementary Figure 1.** The correlation analysis of methane and environmental substrates (Digits represents Pearson correlation coefficients; filled color indicates significant correlation, blue represents positive correlation, red represents negative correlation, shade of color represents strength of correlation.

**Supplementary Figure 2.** Relative abundance of archaeal families classified into *Bathyarchaeota*, *Thaumarchaeota* or *Euryarchaeota* (a), and bacteria at genus level (b).

**Supplementary Figure 3.** The relationship between bacterial community and shallow gas. The difference of α-diversity indexes (a) and relative abundance of bacterial genus (b) between gas-bearing layers and gas-free layers.


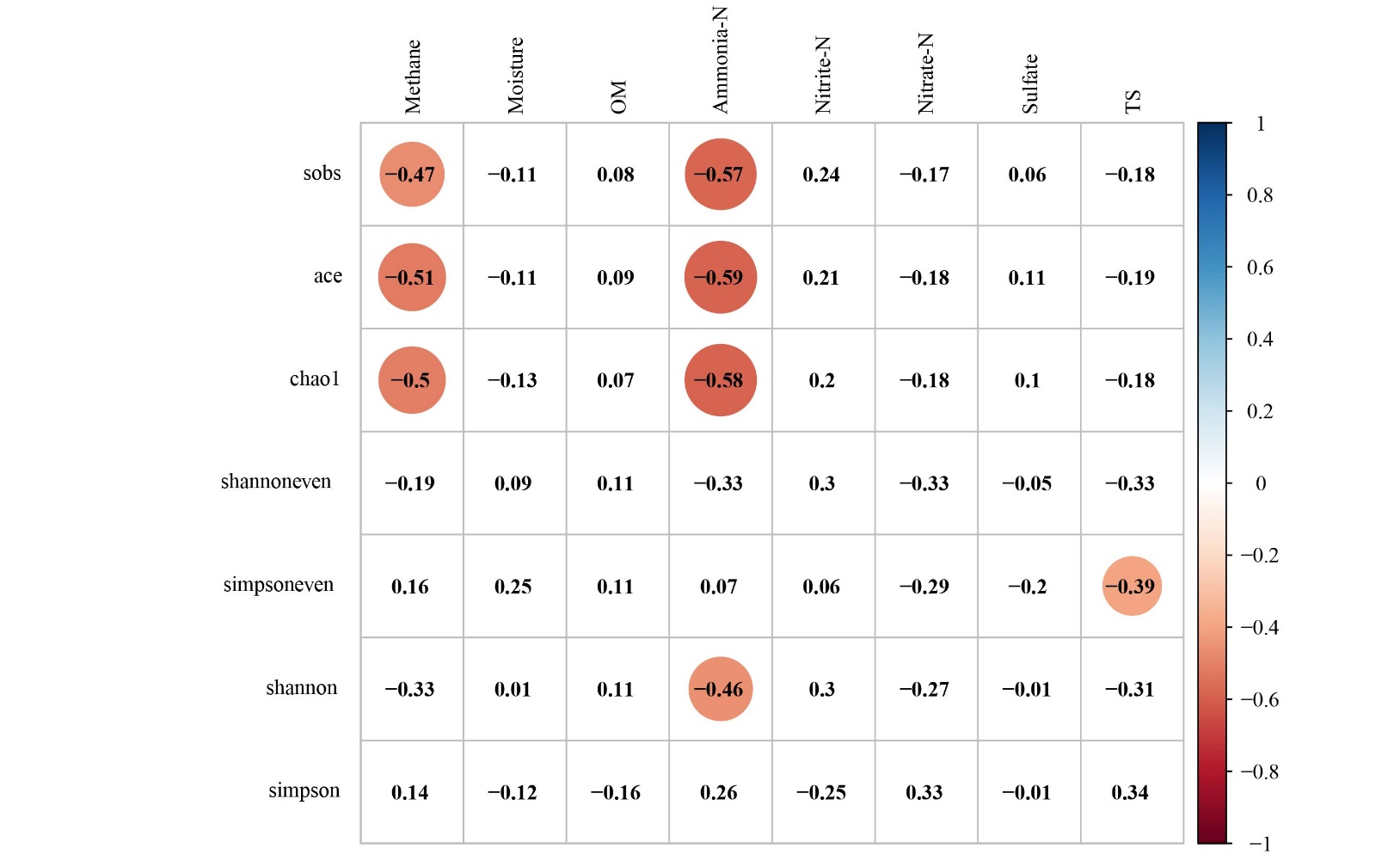


**Supplementary Figure 4.** The correlation analysis between the α-diversity of bacterial community, methane and environmental substrates.


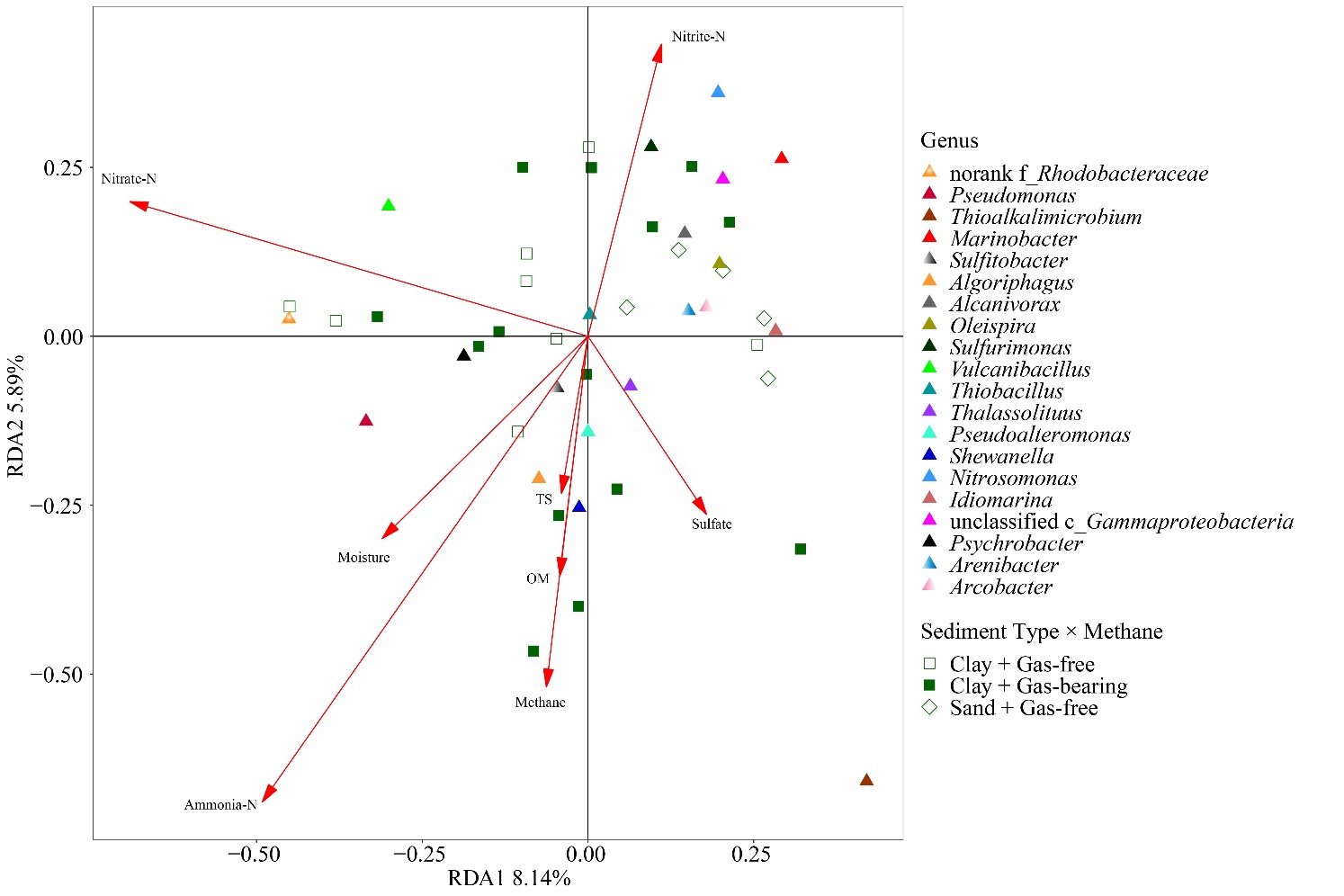


**Supplementary Figure 5** Ordination plot of redundancy analysis (RDA) for the dominant bacterial genera with methane and environmental substrates as constraining variables.

**Supplementary Figure 6** Analysis of biogas potential of the sediment samples in Yushan Island.

**Supplementary Figure 7** Environmental substrates in different strata. The difference of environmental substrates between gas-bearing layers and gas-free layers (a), and between clay strata and sand strata.


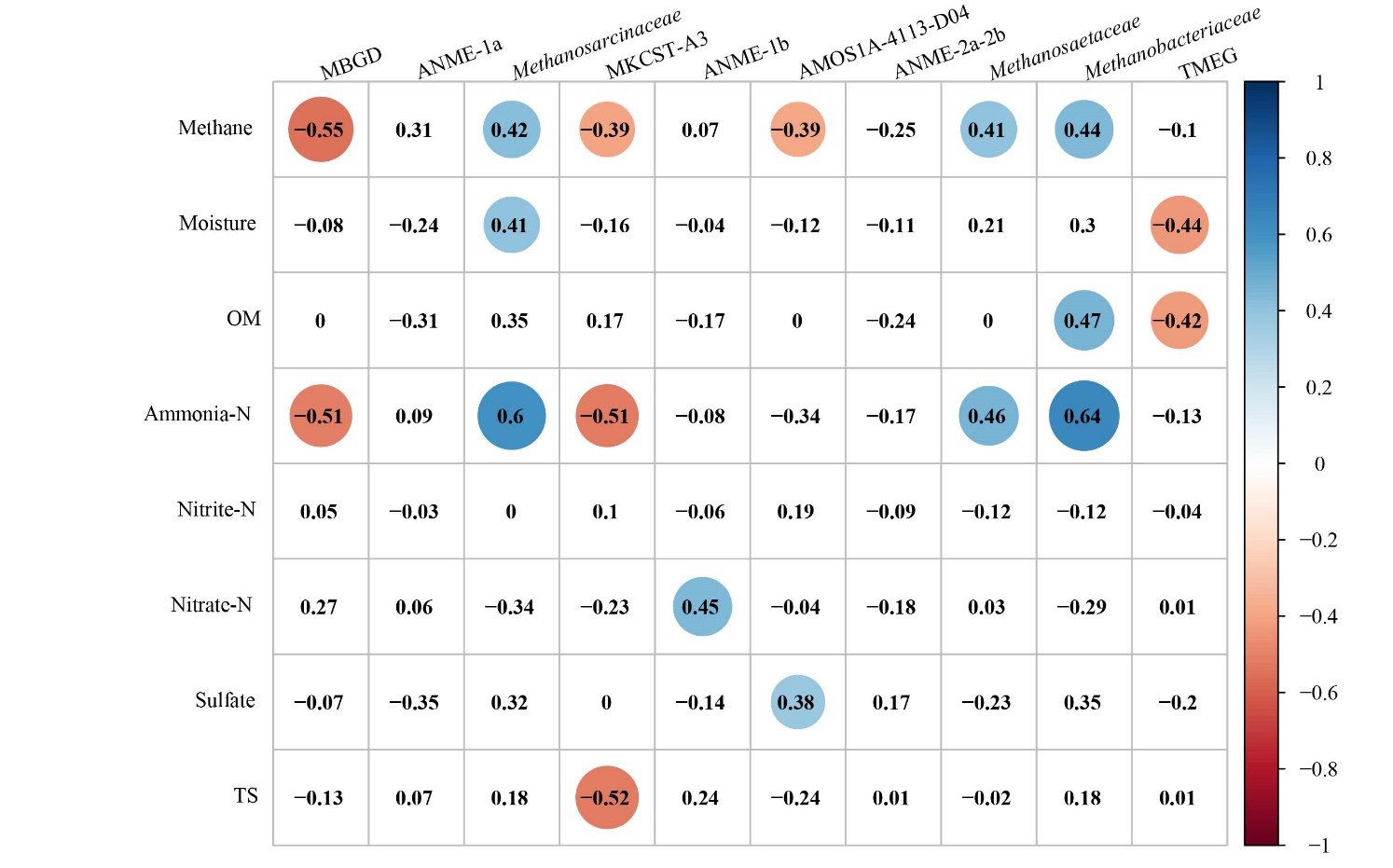


**Supplementary Figure 8** The correlation analysis for the dominant archaeal families with methane and environmental substrates.


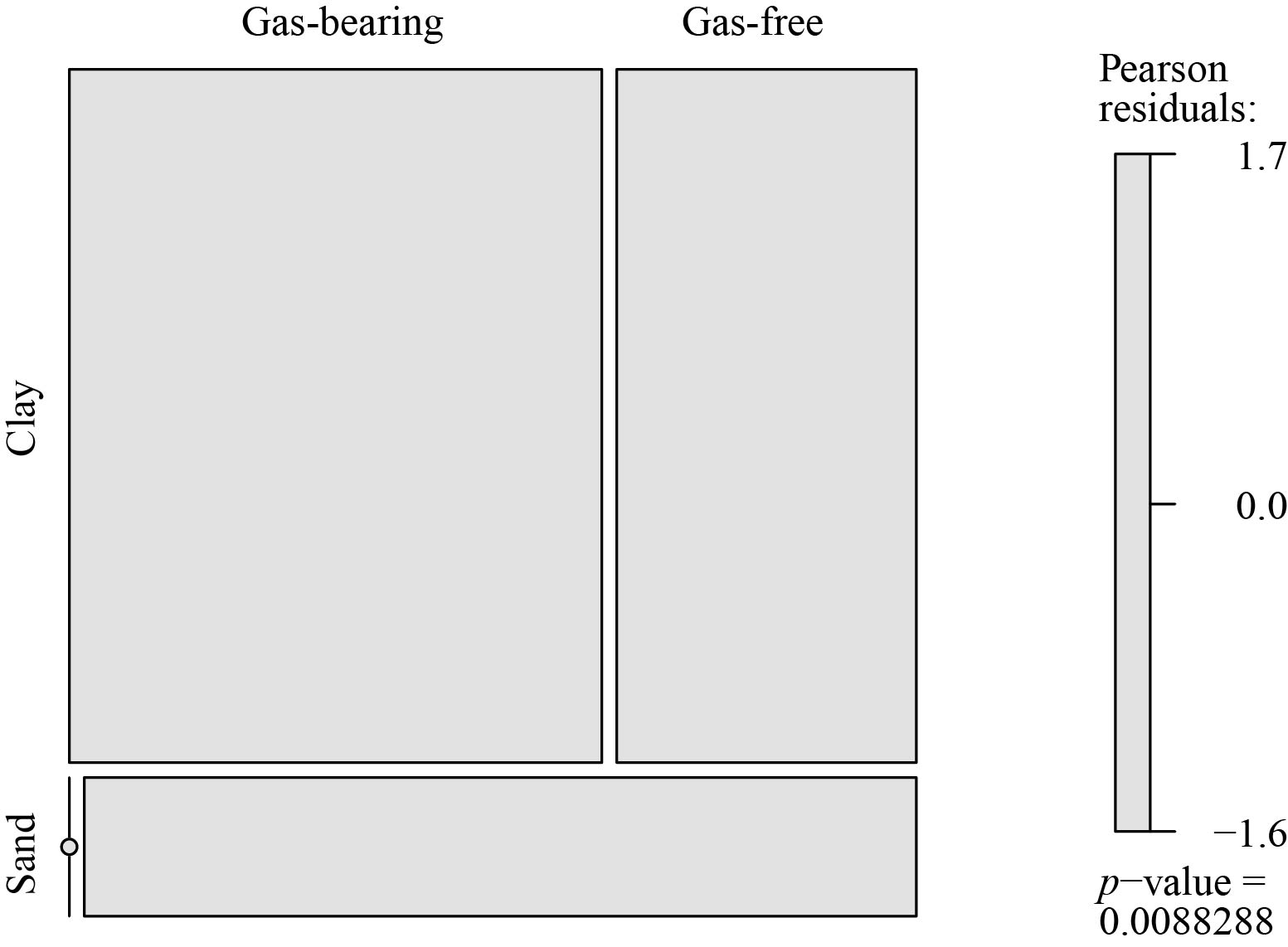


**Supplementary Figure 9** Chi-square test of shallow gas and sediment type (mosaic plot, sample size was shown as rectangular area, Pearson residuals were shown as color).

## Supplementary Tables

**Table S1** Sampling position

| Site | Sampling date | North latitude | East longitude |
| --- | --- | --- | --- |
| YS1 | 2017.3.26 | 30°21′31.21″ | 121°53′12.56″ |
| YS3 | 2017.3.12 | 30°20′18.95″ | 121°53′08.55″ |
| YS4 | 2017.3.30 | 30°20′45.32″ | 121°54′09.64″ |
| YS5 | 2017.4.1 | 30°19′13.98″ | 121°55′17.34″ |
| YS7 | 2017.3.29 | 30°20′12.23″ | 121°55′16.91″ |
| YS8 | 2017.3.8 | 30°17′47.21″ | 121°58′49.87″ |

**Table S2** Environmental substrates and grouping basis

| ID | Depth, m | Sediment type | Methane, g/m^3^ | | Moisture, % | OM, g/kg-DW | Ammonia-N, g/kg-WW | Nitrite-N, g/kg-WW | Nitrate-N, g/kg-WW | Sulfate, g/kg-DW | TC, g/kg-DW | TN, g/kg-DW | TS, g/kg-DW |
| --- | --- | --- | --- | --- | --- | --- | --- | --- | --- | --- | --- | --- | --- |
| YS1-1 | -1.5 | Clay | | 0.0 | 3.22 | 6.881 | 0.132 | 0.000 | 0.009 | 0.201 | 9.1892 | 0.2738 | 0.5652 |
| YS1-3 | -4.5 | Clay | | 4.0 | 3.14 | 5.313 | 0.110 | 0.019 | 0.005 | 0.117 | 8.1718 | 0.2065 | 0.4842 |
| YS1-5 | -6.5 | Clay | | 7.6 | 3.56 | 5.203 | 0.277 | 0.000 | 0.005 | 0.107 | 8.6769 | 0.2774 | 0.5278 |
| YS1-8 | -10.5 | Clay | | 11.3 | 3.40 | 5.783 | 0.208 | 0.003 | 0.006 | 0.176 | 8.9444 | 0.3033 | 0.5036 |
| YS1-11 | -14.0 | Clay | | 0.0 | 4.16 | 6.244 | 0.225 | 0.000 | 0.007 | 0.454 | 8.5426 | 0.3287 | 0.4653 |
| YS1-16 | -19.0 | Clay | | 0.0 | 3.87 | 7.403 | 0.200 | 0.000 | 0.005 | 0.580 | 8.4838 | 0.3428 | 0.4494 |
| YS1-18 | -21.5 | Clay | | 2.1 | 3.36 | 4.392 | 0.037 | 0.013 | 0.003 | 0.356 | 7.0248 | 0.1821 | 0.4248 |
| YS3-2 | -3.0 | Clay | | 8.5 | 3.42 | 4.897 | 0.245 | 0.000 | 0.003 | 0.272 | 8.0871 | 0.2526 | 0.4633 |
| YS3-6 | -7.7 | Clay | | 16.5 | 3.64 | 6.623 | 0.399 | 0.000 | 0.004 | 0.304 | 9.3985 | 0.3689 | 0.4805 |
| YS3-16 | -19.1 | Clay | | 11.0 | 3.04 | 6.130 | 0.345 | 0.000 | 0.003 | 0.221 | 9.0562 | 0.3651 | 0.4208 |
| YS3-20 | -23.4 | Clay | | 3.7 | 1.85 | 1.614 | 0.124 | 0.000 | 0.003 | 0.142 | 1.0331 | 0.1177 | 0.4396 |
| YS4-3 | -5.0 | Clay | | 0.0 | 3.78 | 6.057 | 0.191 | 0.001 | 0.003 | 0.342 | 11.4540 | 0.3575 | 0.2697 |
| YS4-9 | -11.0 | Clay | | 0.0 | 5.13 | 8.404 | 0.261 | 0.003 | 0.002 | 0.452 | 9.8650 | 0.3716 | 0.2741 |
| YS4-17 | -19.0 | Clay | | 0.0 | 3.81 | 6.776 | 0.132 | 0.000 | 0.003 | 0.616 | 8.3031 | 0.2225 | 0.1081 |
| YS4-27 | -30.0 | Clay | | 14.0 | 4.09 | 4.652 | 0.579 | 0.002 | 0.002 | 0.262 | 6.3421 | 0.1882 | 0.1783 |
| YS5-1 | -1.5 | Clay | | 1.5 | 4.25 | 8.316 | 0.039 | 0.000 | 0.002 | 0.328 | 12.7352 | 0.4167 | 0.1107 |
| YS5-8 | -10.0 | Clay | | 5.5 | 3.92 | 8.848 | 0.077 | 0.001 | 0.003 | 0.255 | 11.6504 | 0.3580 | 0.0817 |
| YS5-11 | -14.0 | Clay | | 0.0 | 2.76 | 9.379 | 0.010 | 0.000 | 0.003 | 0.261 | 9.5342 | 0.2869 | 0.1774 |
| YS5-18 | -23.0 | Sand | | 0.0 | 3.23 | 6.003 | 0.004 | 0.000 | 0.002 | 0.942 | 6.5808 | 0.1814 | 0.3064 |
| YS7-3 | -4.3 | Clay | | 0.0 | 4.59 | 7.841 | 0.032 | 0.008 | 0.002 | 0.457 | 10.6519 | 0.3060 | 0.2646 |
| YS7-8 | -10.0 | Sand | | 0.0 | 3.18 | 4.028 | 0.020 | 0.000 | 0.003 | 0.124 | 6.7221 | 0.1332 | 0.6776 |
| YS7-14 | -17.0 | Sand | | 0.0 | 1.99 | 1.713 | 0.029 | 0.000 | 0.002 | 0.481 | 0.7887 | 0.0862 | 0.5627 |
| YS7-26 | -30.5 | Clay | | 0.0 | 4.40 | 8.275 | 0.115 | 0.032 | 0.004 | 0.464 | 8.8093 | 0.3625 | 0.4357 |
| YS7-46 | -55.0 | Sand | | 0.0 | 2.93 | 6.017 | 0.013 | 0.001 | 0.005 | 0.400 | 7.3418 | 0.2235 | 0.4187 |
| YS8-9 | -10.6 | Sand | | 0.0 | 1.94 | 3.777 | 0.055 | 0.012 | 0.002 | 0.239 | 5.4276 | 0.1361 | 0.6042 |
| YS8-12 | -14.0 | Clay | | 24.3 | 3.42 | 6.562 | 0.204 | 0.000 | 0.002 | 0.466 | 7.5136 | 0.1320 | 0.5638 |
| YS8-17 | -19.0 | Clay | | 14.4 | 4.67 | 7.691 | 0.555 | 0.000 | 0.002 | 0.491 | 9.4387 | 0.4044 | 0.4715 |
| YS8-21 | -24.0 | Clay | | 14.1 | 4.64 | 8.711 | 0.683 | 0.003 | 0.002 | 0.447 | 9.7954 | 0.4724 | 0.4640 |
| YS8-26 | -29.0 | Clay | | 13.4 | 4.09 | 9.561 | 0.676 | 0.000 | 0.002 | 0.724 | 9.1788 | 0.4734 | 0.5495 |
| YS8-30 | -35.0 | Clay | | 30.1 | 4.65 | 10.517 | 0.922 | 0.000 | 0.002 | 0.419 | 10.8667 | 0.7536 | 0.4835 |

| **Table S3** Results of redundancy analysis of top 10 archaeal families | | | | |
| --- | --- | --- | --- | --- |
| Environmental factors | RDA1 | RDA2 | r^2^ | *p* |
| Methane | 0.95799 | 0.2868 | 0.3842 | 0.002 |
| OM | 0.96591 | -0.25886 | 0.3048 | 0.008 |
| Moisture | 0.98747 | 0.15781 | 0.2367 | 0.029 |
| Ammonia-N | 0.95119 | 0.30861 | 0.36933 | 0.001 |
| Nitrite-N | -0.8179 | 0.57535 | 0.026 | 0.737 |
| Nitrate-N | -0.89101 | 0.45398 | 0.2389 | 0.037 |
| Sulfate | 0.99917 | -0.04064 | 0.1345 | 0.142 |
| TS | 0.84733 | 0.53106 | 0.057 | 0.47 |
| RDA1, RDA2: Scores of environmental factors on two axes. r^2^: The determining coefficients of environmental factors on variation of microbial community. *p*: level of significance | | | | |

| **Table S4** Results of redundancy analysis of top 20 bacterial genera | | | | |
| --- | --- | --- | --- | --- |
| Environmental factors | RDA1 | RDA2 | r^2^ | *p* |
| Depth | -0.01141 | -0.99993 | 0.3854 | 0.008 |
| Methane | 0.05809 | -0.99831 | 0.0741 | 0.406 |
| OM | -0.62319 | -0.78207 | 0.1318 | 0.194 |
| Moisture | -0.43609 | -0.8999 | 0.5272 | 0.001 |
| Ammonia-N | 0.20823 | 0.97808 | 0.1604 | 0.108 |
| Nitrite-N | -0.94134 | 0.33745 | 0.3205 | 0.007 |
| Nitrate-N | 0.48033 | -0.87709 | 0.0695 | 0.435 |
| Sulfate | -0.08381 | -0.99648 | 0.0387 | 0.622 |
| TS | -0.01141 | -0.99993 | 0.3854 | 0.008 |
| RDA1, RDA2: Scores of environmental factors on two axes. r^2^: The determining coefficients of environmental factors on variation of microbial community. *p*: level of significance | | | | |
